# The Impact of Hip Arthroscopy on the Progression of Hip Osteoarthritis in Patients With Femoroacetabular Impingement Syndrome: A Systematic Review and Meta-analysis

**DOI:** 10.1177/23259671251326116

**Published:** 2025-04-02

**Authors:** Darius L. Lameire, Ananya Pathak, Shu Yang Hu, Yue Ting Kero Yuen, Daniel B. Whelan, Tim Dwyer, Tyler M. Hauer, Jaskarndip Chahal

**Affiliations:** † Division of Orthopaedic Surgery, University of Toronto, Toronto, Ontario, Canada; ‡ Division of Orthopaedic Surgery, Women’s College Hospital, Toronto, Ontario, Canada; § University of Toronto Orthopaedic Sports Medicine, Toronto, Ontario, Canada; ‖ Division of Orthopaedic Surgery, St. Michael’s Hospital, Toronto, Ontario, Canada; ¶ Division of Orthopaedic Surgery, Mount Sinai Hospital, Toronto, Ontario, Canada; Investigation performed at the Division of Orthopaedic Surgery, Department of Surgery, University of Toronto, Ontario, Canada

**Keywords:** arthroscopy, femoroacetabular, femoroacetabular impingement syndrome, hip, impingement, osteoarthritis

## Abstract

**Background:**

Hip arthroscopy (HA) for the surgical management of femoroacetabular impingement syndrome (FAIS) provides reliable improvements in pain and function; however, debate remains regarding the impact of HA on the progression of osteoarthritis (OA).

**Purpose:**

To determine whether HA for FAIS reduces the progression of OA and the risk of conversion to total hip arthroplasty (THA).

**Study Design:**

Systematic review; level of evidence, 4.

**Methods:**

A systematic electronic search of articles in Medline, Embase, and ClinicalTrials.gov databases was performed under the PRISMA (Preferred Reporting Items for Systematic Reviews and Meta-Analyses) guidelines, with 5046 articles remaining after duplicates were removed. All papers addressing HA for FAIS that reported radiographic progression of hip OA with a follow-up of ≥2 years were eligible for inclusion. Studies assessing labral reconstruction, revision HA, case reports, studies with <10 patients, and patients with hip dysplasia or rheumatoid arthritis were excluded. A total of 322 studies progressed to full text, and 16 studies were ultimately included in this review. Studies were divided based on short-term (ST) (2 to <5 years), mid-term (MT) (5 to <10 years), and long-term (LT) (>10 years) follow-ups. A meta-analysis of homogenous studies and outcomes was performed, otherwise, descriptive statistics were presented.

**Results:**

Sixteen studies (2278 hips) with FAIS were included, in which 1196 hips underwent HA and 1082 hips were treated nonoperatively. There were 8 ST studies, 4 MT studies, and 4 LT studies. A meta-analysis of 2 comparative studies found 32% (*P* = .002) less risk of progression of radiographic OA (any increase in grading) with HA compared with nonoperative management. In addition, there was a nonsignificant 23% (*P* = .35) decreased risk of conversion to THA/hip resurfacing with HA. For all studies, there was a progression of hip OA ranging from 0% to 37.1% for ST studies, 11.5% to 23% for MT studies, and 4.3% to 28% for LT studies.

**Conclusion:**

Our systematic review demonstrated that studies of patients undergoing HA for FAIS demonstrated increased radiographic progression of hip OA over time. Although significantly limited by only 2 retrospective cohort studies, subgroup analysis comparing operative versus nonoperative management demonstrated a 32% reduction in the radiographic progression of OA (any increase in grading) at the LT follow-up. However, there were no significant differences in the risk of THA/hip resurfacing. Future long-term, high-level controlled studies are needed to help further understand this important clinical question.

Femoroacetabular impingement syndrome (FAIS) is the premature contact between the femoral head-neck junction and the acetabular rim.^12^ Whether in the form of cam and/or pincer impingement, along with its associated labral tears, FAIS is becoming increasingly recognized as a significant source of pain for patients, and more hip arthroscopy (HA) is being done each year for this indication.^14,32^ Although both cam and pincer FAIS can cause labral tears, pincer-type impingement typically causes intrasubstance tearing due to repetitive pinching, whereas cam-type impingement typically leads to injuries of the chondrolabral junction.^1^ Patients with cam morphology FAIS exhibit altered biomechanics—including reduced sagittal hip range of motion, abduction angles, hip flexor force, and slower walking speeds.^47^ In addition, studies have shown that the contact pressures on the acetabular cartilage are significantly elevated in patients with cam FAIS and have elevated shear stresses.^31^ As such, these altered hip biomechanics and increased joint contact forces may lead to progressive cartilage wear and hip osteoarthritis (OA).^20^ Several authors have reported excellent results and high patient satisfaction after arthroscopic treatment of FAIS.^7,25,33^ For example, in their meta-analysis of 81 studies, Levy et al^24^ showed clinically significant improvements in patient-reported outcomes of Harris Hip Scores and Hip Outcome scores after HA in >90% of studies at 31 ± 20 months. Therefore, one goal of HA is to create a favorable intra-articular environment to potentially prevent further joint damage and reduce functional pain.^42^

OA is the 15th highest cause of years lived with disability worldwide, with hip OA being the third leading contributor to this.^23^ We now understand that the cause of hip OA is multifactorial and may include the presence of FAIS. This is not a new concept, Ganz et al^9^ had discussed FAIS and a novel surgical approach in 2001, and linked FAIS with OA in^10^ 2003. In a systematic review by Kowalczuk et al,^20^ FAIS and, in particular, cam morphology with elevated alpha angles of ≥60° were shown to predispose patients to the development of hip OA. However, the connection between pincer morphology and increased development of OA is less clear.^19,20^ In theory, the arthroscopic management of FAIS could potentially have significant effects on reducing the burden of disease related to OA. Although much research has assessed the risk of progression to total hip arthroplasty (THA) after HA, the progression of radiographic hip OA has not been investigated to the same degree.^28^ There have been no recent systematic reviews or meta-analyses to date examining how hip OA progresses after HA for the treatment of FAIS, as well as the impact it may have on the natural history of hip OA for patients with FAIS.

Multiple recent comparative studies have been published assessing the impact HA may have on the progression of hip OA compared with nonoperative management, and there have been indications that there may be a decrease in OA progression with a long-term (LT) follow-up.^17,37^ Therefore, this systematic review aimed to determine whether HA for FAIS may reduce the progression of OA and, secondarily, the risk of conversion to THA. We hypothesized that patients who underwent HA for FAIS would have a reduced risk of OA progression and higher survivorship (lower conversion to THA) compared with their peers who did not receive the treatment.

## Methods

This systematic review focused on radiographic outcomes of hip OA for patients undergoing HA for FAIS. This review followed the PRISMA (Preferred Reporting Items for Systematic Reviews and Meta-Analysis) algorithm and guidelines.^29^ The study was registered in PROSPERO before data abstraction (CRD42024514523).

### Comprehensive Search Strategy

A systematic search of 3 databases (Embase, Medline, and ClinicalTrials.gov), combined with a manual search, was performed through March 10, 2024, by 2 reviewers (D.L.L. and A.P.) for the literature related to HA in the context of FAIS and radiographic OA. The complete search strategies can be found in Supplemental Content 1. The inclusion criteria for this review were as follows: (1) adult population (mean age, ≥18 years); (2) patients with symptomatic FAIS; (3) reported OA based on radiographic assessment via Tönnis or Kellgren-Lawrence (K-L) grading; (4) any grade of preoperative OA; (5) OA progression reported or follow-up radiographic OA grading provided; (6) follow-up of ≥2 years; (7) HA with concomitant labral repair/debridement and/or femoral/acetabular osteochondroplasty; (8) all levels of evidence; and (9) full-text available in English. The exclusion criteria were (1) labral reconstruction to limit heterogeneity; (2) diagnostic or revision HA; (3) case reports or case series with <10 patients; (4) patients with hip dysplasia (lateral center-edge angle less than 20 degrees or as defined by authors) or rheumatoid arthritis; (5) concomitant periacetabular osteotomy; and (6) patients undergoing HA for pathologies other than FAIS.

### Study Screening

Two authors (D.L.L. and A.P.) independently assessed the title and abstract of all studies. All studies with insufficient data, as well as any disagreement between authors, were advanced to the full-text review stage. After an independent dual review of the full text, a third author (T.M.H.) resolved any disagreements. A Kappa (κ) score was used to evaluate the level of agreement between reviewers.^21^ The Methodological Index for Non-randomized Studies (MINORS) was used to assess the quality of studies.^43^ The maximum score based on MINORS is 24 and 16 for comparative and noncomparative studies, respectively. The risk of bias (ROB) grade was reported based on previously published criteria.^20^ The Cochrane Collaboration’s tool for assessing the ROB in randomized trials was used to assess the methodological quality of the randomized control trials.^44^

### Data Abstraction

Two reviewers (D.L.L. and A.P.) abstracted the data from each study with predetermined tables using Google Sheets (Alphabet, Inc). The data abstracted were as follows: study characteristics (authors, study design, journal, publication year, level of evidence, etc); number of patients and/or hips; descriptive data (age, sex, body mass index, etc); follow-up; radiographic parameters (Tönnis grade, lateral center-edge angle, alpha angle, etc); conversions to THA/hip resurfacing; revision HA; risk factors for progression of OA; and subjective outcomes. The level of evidence for all studies was determined based on the author’s reporting, or if not stated, based on the AAOS Evidence-Based Practice Committee guidelines.^46^

### Primary Outcomes

The primary outcome of this study was a radiographic progression of hip OA. This was determined as reported by papers or as determined by changes in Tönnis or KL grading at the follow-up. Overall radiographic OA grades were also averaged and compared pre- and postoperatively. If studies reported the progression of OA but excluded patients who underwent THA before the follow-up radiographic assessment, they were graded as Tönnis grade 3 (highest possible grade) so that these patients would be accounted for in the overall progression of OA.

### Secondary Outcomes

The secondary outcomes consisted of conversion to THA, revision HA, patient-reported outcome measures (PROMs), and risk factors for the progression of OA. Conversion to THA/hip resurfacing or revision HA was determined as the percentage of patients who went on to have a THA/hip resurfacing or repeat HA, respectively, within the follow-up period. Abstracted PROMs included the modified Harris Hip Score (mHHS), Hip Outcome Score (HOS), Non-Arthritic Hip Score, visual analog score for pain (VAS-Pain), and patient satisfaction scores.

### Follow-up Duration

Studies were stratified based on their mean follow-up or their minimum follow-up if a mean was not available. They were divided into 3 follow-up periods: short-term (ST) (2 to <5 years), mid-term (MT) (5 to <10 years), and LT (≥10).

### Subgroup Analysis

Studies comparing operative and nonoperative outcomes of FAIS were analyzed as a subgroup.

### Statistical Analysis

For comparative studies assessing the operative versus nonoperative management of FAIS, a meta-analysis was performed using RevMan (The Cochrane Collaboration)^38^ to determine risk ratios (RRs). The Mantel-Haenszel method was used using a random effects model. Weighted means were derived using Google Sheets (Alphabet, Inc). Frequency-weighted means were calculated by comparing the value of the outcome of interest from each study and then calculating the relative weighting of that score based on the number of patients included in the study. Otherwise, outcomes were presented in a narrative summary fashion.

## Results

**Figure 1.**
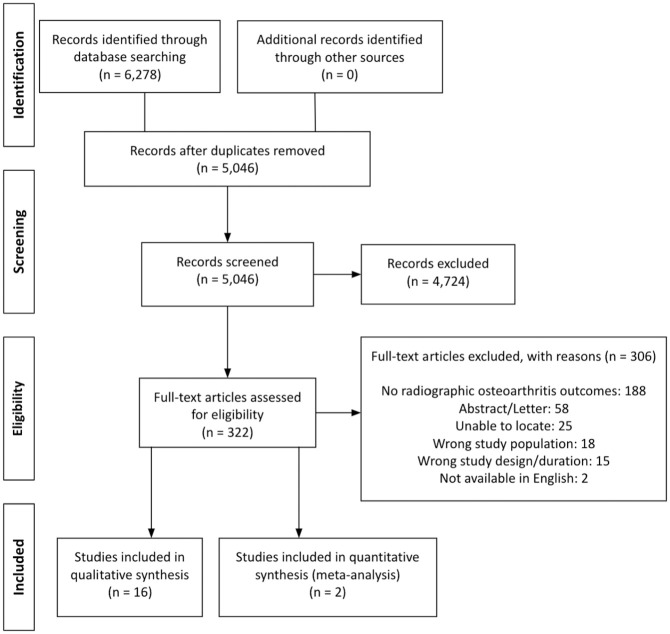
PRISMA Diagram. PRISMA, Preferred Reporting Items for Systematic Reviews and Meta-Analyses.

A total of 6278 articles were identified from the initial search, with 5046 remaining after duplicates were removed. All studies were assessed in full text, and 16 articles remained after the application of the inclusion and exclusion criteria (Figure 1).^#^ Study characteristics can be found in Supplemental Content 2. Title and abstract screening, as well as full-text screening, both had substantial agreement (κ = 0.766 [95% CI, 0.725-0.807] and κ = 0.807 [95% CI, 0.641-0.972]), respectively. There were 6 comparative studies. This included 1 randomized controlled trial (RCT)^39^ (level 2 evidence) and 5 cohort studies^3,11,17,35,37^ (4 level 3 and 1 level 4 evidence). Of note, 1 study^11^ compared outcomes of patients with Tönnis grade 0 and 1, and therefore, outcomes were combined for the majority of reporting. Also, 10 noncomparative case series studies^**^ were included (all level 4 evidence). The ROB assessment can be found in Supplemental Content 3. The MINORS assessment score for the non-RCT comparative studies ranged from 20 to 22 (mean, 20.6; 3 good and 2 excellent methodological quality), and the MINORS score for all noncomparative studies ranged from 12 to 15 (mean, 12.9; 8 good and 2 excellent methodological quality; [Supplemental Content 2]). Two comparative studies^17,37^ compared HA to nonoperative management of FAIS, both of which had LT follow-ups.

### Patient Characteristics

There were a total of 2278 hips (2103 patients) with FAIS included in this analysis. This included 1196 hips (1168 patients) that underwent HA and 1082 hips (935 patients) that were managed nonoperatively (Table 1). The mean age ranged from 19.3 to 44.2 years. For operative patients, the percentage of female patients ranged from 18.9% to 92%, the mean body mass index (BMI) ranged from 21.7 to 34.3 kg/m^2^, and FAIS diagnosis ranged from 17% to 100% isolated cam morphology, 4.4% to 25% isolated pincer morphology, and 5.6% to 70.5% combined morphology. Eight studies^7,11,15,16,25,33,34,39^ had an ST follow-up, with a mean of 2.3 to 4.6 years of follow-up (1 study only reported the range, with a minimum 3-year follow-up), 4 studies^13,18,22,35^ with MT follow-ups ranged from a minimum of 5 to a mean of 7.7 years of follow-up, and 4 studies^3,17,26,37^ with LT follow-ups ranged from a minimum of 10 to a mean of 12.8 years of follow-up.

**Table 1:** Patient Characteristics^*a*^

		Participants			Age		Follow-up		Side		BMI		FAIS Pathology		
Author (Year)		Patients, n	Hips, n	Female, %	Years	SD (Range)	Years	SD (Range)	Right	Left	BMI kg/m^2^	SD (Range)	Cam, %	Pincer, %	Combo, %
*Short-term*															
Domb et al^7^ (2013)		22	22	81.8	20	NR (14-39)	2.3	0.5 (1.4-3.3)	11	11					
Gicquel et al^11^ (2014)		51	53	62.7	31	NR	4.6	4.2-5.5	31	22			43	19	38
Hartigan et al^15^ (2016)		78	82	67.9	23.4	NR (15-40)	3.2	NR (1.8-6.5)	50	32	23	NR (18-34)			
Hufeland et al^16^ (2016)		44	44	45.5	34.3	13.2 (17-65)	3.1	1.2 (3.6-7.3)							
Mardones et al^25^ (2016)		15	17	73.3	33.5	10.7 (18-49)	4	NR					17	12.5	70.5
Palmer et al^33^ (2012)		185	201	50.7	40.2	NR (14-87)	3.8	NR	119	82			75.6		24.4
Perets et al^34^ (2017)		39	41	79.5	19.3	4.7 (NR)	2.8	1(2-5.4)	19	22	21.7	4.2 (NR)			
Rhee et al^39^ (2016)															
	HA, knot-tying	19	19	47.4	33.8	11.8 (NR)	2.7	2.1-3.4	10	9	22.4	2.4 (NR)	21	21	15.8
	HA, knotless	18	18	72.2	34.6	11.8 (NR)	2.7	2.2-3.4	12	6	22.1	3.6 (NR)	22.2	11.1	5.6
*Mid-term*															
Haefeli et al^13^ (2017)		50	52	92	35	12 (16-63)	6.6	1.3 (5-10.7)	34	16	24	5 (18-38)	48	25	27
Kierkegaard et al^18^ (2022)		60	60	63.3	36	9 (NR)	NR	Min, 5y							
Lee et al^22^ (2019)		41	41	48.8	34.6	NR (16-54)	7.7	NR (7.1-9.8)	26	15	24.3	3.3 (19-39)			
Perets et al^35^ (2018)															
	HA, obesity	74	74	60.8	44.2	11.6 (19-75)	6	0.9 (5-8.6)			34.3	3.9 (30-49)			
	HA, control	74	74	60.8	44.2	11.8 (80-72)	5.9	0.8 (5-7.7)			22.7	1.6 (19-25)			
*Long-term*															
Domb et al^3^ (2023)															
	HA, repair	46	46	60.9	38.7	13 (35-43)	NR	Min, 10y			25.1	4.2 (24-26)			
	HA, debridement	46	46	56.5	42.1	14.9 (38-46)	NR	Min, 10y			25.6	5 (24-27)			
Husen et al^17^ (2023)															
	HA	132	132	64.4	28.2	7.9 (13-41)	10.6	4 (5.2-23.4)	75	57					
	Nonop	835	982	63.7	28.1	9 (7-47)	12.8	4.7 (5.0-23.3)	463	519					
Más Martínez et al^26^ (2024)		74	74	18.9	42	10.7 (NR)	11	0.8 (10-12)			25.2	2.9 (NR)	64.7	4.4	30.9
Ramkumar et al^37^ (2024)															
	HA	100	100	46	33.8	NR (17-53)	12	NR (Min, 10y)			22.5				
	Nonop	100	100	46	33.8	NR (17-53)	12	NR (Min, 10y)			22.5				

a BMI, body mass index; FAIS, femoroacetabular impingement syndrome; HA, hip arthroscopy; Min, minimum; Nonop, nonoperative; NR, not reported; y, years.

### OA Progression

#### Meta-analysis

**Figure 2.**
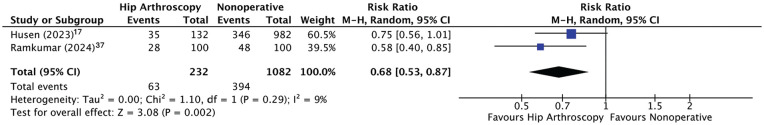
Forest plot demonstrating the risk ratio of hip OA progression with HA versus nonoperative management. HA, hip arthroscopy; M-H, Mantel-Haenszel; OA, osteoarthritis.

Two LT follow-up comparative studies compared the radiographic progression of hip OA between HA and nonoperative management.^17,37^ There was a statistically significant risk reduction for hip OA progression with HA (RR, 0.68 [95% CI, 0.53-0.87]; *P* = .002) (Figure 2). Therefore, patients with HA had 0.68 times the risk of worsening HA compared with nonoperative treatment, or in other words, had a 32% less risk of any progression of radiographic grading of hip OA.

#### All Studies

**Figure 3.**
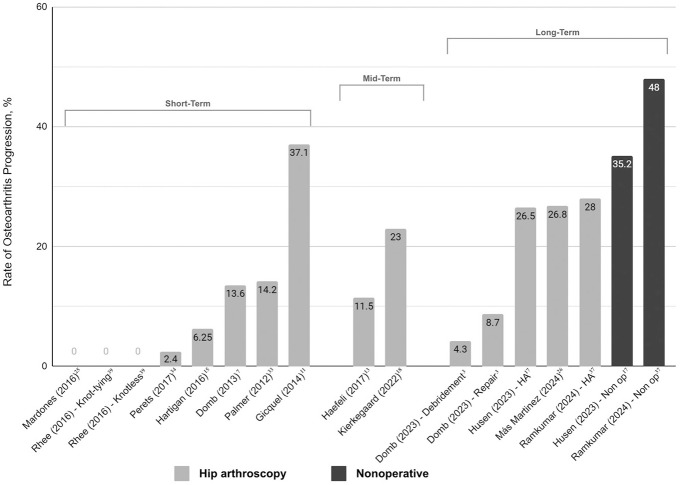
Progression of hip OA. OA, osteoarthritis.

For HA, the reported rate of OA progression at the ST follow-up ranged from 0% to 37.1%; for the MT follow-up group, it ranged from 11.5% to 23%, and for the LT follow-up group, it ranged from 4.3% to 28% (Figure 3). For nonoperative management, reported progression of OA at the LT follow-up ranged from 35.2% to 48%. For the 2 homogenous comparative LT studies,^17,37^ assessing operative and nonoperative treatment, the weighted mean of OA progression for HA was 27.1% (SD, 1%), and it was 36.4% (SD, 3.9%) for nonoperative treatment.

For studies with ST follow-up, the mean reported preoperative baseline Tönnis grade ranged from 0 to 0.41, and it ranged from 0.02 to 0.41 at the follow-up (Table 2). One study^16^ with an ST follow-up used the KL grading system and had a mean baseline of 2, which progressed to 2.2. The mean preoperative baseline Tönnis grade for MT studies ranged from 0.17 to 0.51 and progressed to a range of 0.30 to 0.79. For operative studies with LT follow-up, the mean preoperative baseline Tönnis grade ranged from 0.20 to 1.15, and nonoperative studies’ initial assessment baseline ranged from 0.28 to 0.71. At an LT follow-up, the mean Tönnis grade ranged from 0.28 to 1.30 for operative hips and 0.87 to 1.11 for nonoperative hips.

**Table 2:** Reported Radiographic Osteoarthritis Grades^*a*^

		Baseline							Follow-up						
			Tönnis Grade, n					KL Grade		Tönnis Grade, n					KL Grade
Author (Year)		Hips, n	0	1	2	3	Mean (Range)	Mean (Range)	Hips, n	0	1	2	3	Mean (Range)	Mean (Range)
*Short-term*															
Domb et al^7^ (2013)		22	22^*b*^						22	22^*b*^					
Gicquel et al^11^ (2014)		53	35	18			0.34 (0-1)								
Hartigan et al^15^ (2016)		15	13	2			0.13 (0-1)		16	13	2		1	0.13 (0-1)	
Hufeland et al^16^ (2016)		44						2.0 (0.3)	38						2.2 (0-3)
Mardones et al^25^ (2016)		17	10	7			0.41 (0-1)		17	10	7			0.41 (0-1)	
Palmer et al^33^ (2012)		201	174	25	1		0.13 (0-2)		106						
Perets et al^34^ (2017)		41	41				0 (0)		41	40	1			0.02 (0-1)	
Rhee et al^39^ (2016)															
	HA, knot-tying	19	19^*b*^						19	19^*b*^					
	HA, knotless	18	18^*b*^						18	18^*b*^					
*Mid-term*															
Haefeli et al^13^ (2017)		52	43	9			0.17 (0-1)		31	17	13	1		0.48 (0-2)	
Kierkegaard et al^18^ (2022)		39	29	8	2		0.31 (0-2)		43	20	16	3	4	0.79 (0-3)	
Lee et al^22^ (2019)		41					0.51 (0-1)		41					0.67 (0-3)	
Perets et al^35^ (2018)															
	HA, obesity	74	49	25			0.34 (0-1)		74	47	25	1	1	0.41 (0-3)	
	HA, control	74	52	22			0.30 (0-1)		74	52	22			0.30 (0-1)	
*Long-term*															
Domb et al^3^ (2023)															
	HA, repair	46	37	9			0.20 (0-1)		46	33	13			0.28 (0-1)	
	HA, debridement	46	31	15			0.33 (0-1)		46	29	17			0.37 (0-1)	
Husen et al^17^ (2023)															
	HA	132	43	86	3		0.70 (0-2)		132	27	81	16	8	1.04 (0-3)	
	Nonoperative	982	350	570	56	6	0.71 (0-3)		982	209	507	215	51	1.11 (0-3)	
Más Martínez et al^26^ (2024)		71	23	23	16	9	1.15 (0-3)		71	23	22	8	18	1.30 (0-3)	
Ramkumar et al^37^ (2024)															
	HA	100	78	20	2		0.24 (0-2)		100	57	34	2	7	0.59 (0-3)	
	Nonop	100	73	26	1		0.28 (0-2)		100	38	45	9	8	0.87 (0-3)	

a HA, hip arthroscopy; KL, Kellgren-Lawrence; Nonop, nonoperative.

b Denotes grade 0 or 1.

The proportion of worsened Tönnis grades was assessed for all studies that reported baseline and follow-up Tönnis grades (Supplemental Digital Content 4). For ST follow-ups (all operative), the proportion of worsened Tönnis grades ranged from 0% to 6.3%. For MT follow-ups, the proportion ranged from 0% to 27.9%. For operative management at LT follow-ups, the proportion of worsened Tönnis grade ranged from 4.3% to 21%, and for nonoperative, it ranged from 20.8% to 35%.

### Risk Factors for OA Progression

One study^11^ found a statistically significant risk of OA progression for patients with baseline Tönnis grade 1 OA (8/14; 57%) compared with Tönnis grade 0 OA (5/21; 24%; *P* = .046). However, they did not find any statistically significant relationships between preoperative offset, alpha angle, lateral center edge angle, or anterior center edge angle. In addition, 1 study^37^ found an absolute risk reduction of OA progression of 20% (relative risk reduction, 42%) with HA compared with nonoperative management. OA progression assessment based on progressive baseline and follow-up/postoperative alpha angles can be found in Table 3. No other studies reported specific risk factors for OA progression.

**Table 3:** Progression of OA and Conversion Rates Based on the Alpha Angle^*a*^

	Alpha Angle				
Author (Year)	Degrees	SD (Range)	Follow-up	Progression of OA, %	Conversion to THA/Hip Resurfacing, %
*Baseline*					
Perets et al^34^ (2017)	54.9	10.7 (NR)	ST	2.4	
Haefeli et al^13^(2017)	59	11 (42-79)	MT	11.5	3.8
Perets et al^35^ (2018), control	59.3	12 (NR)	MT	0	14.9
Mardones et al^25^ (2016)	60	NR (NR)	ST	0	
Hartigan et al^15^ (2016)	60.2	12.1 (NR)	ST	6.3	6.3
Domb et al^3^ (2023), labral repair	61	13 (57.2-64.7)	LT	8.7	10.9
Domb et al^3^ (2023), labral debridement	61.5	11.6 (58.1-64.8)	LT	4.3	21.7
Husen et al^17^ (2023), nonoperative	61.9	14.5 (28.9 - 99.6)	LT	35.2	10.5
Perets et al^35^(2018), obesity	62.1	9.9 (NR)	MT	2.7	29.7
Husen et al^17^(2023), HA	63.9	13.6 (31.6 - 97.8)	LT	26.5	6.8
Más Martínez et al^26^ (2024)	63.9	8.7 (NR)	LT	26.8	18.3
Palmer et al^33^ (2012)	72.3	17 (NR)	ST	14.2	7.5
*Follow-up*					
Perets et al^34^ (2017)	42.7	6.4 (NR)	ST	2.4	
Más Martínez et al^26^ (2024)	43.3	7.8 (NR)	LT	26.8	18.3
Haefeli et al^13^ (2017)	44	8 (32-72)	MT	11.5	3.8
Perets et al^35^ (2018), control	45.3	8.7 (NR)	MT	0	14.9
Hartigan et al^15^ (2016)	45.6	9.2 (NR)	ST	6.3	6.3
Domb et al^3^ (2023), labral repair	46.4	7.5 (44.2-48.5)	LT	8.7	10.9
Mardones et al^25^ (2016)	46.5	NR (NR)	ST	0	
Domb et al^3^ (2023), labral debridement	46.9	9.5 (44.2-49.7)	LT	4.3	21.7
Perets et al^35^ (2018), obesity	50.2	10.5 (NR)	MT	2.7	29.7
Palmer et al^33^ (2012)	53.1	14.4 (NR)	ST	14.2	7.5
Husen et al^17^ (2023), nonoperative	61.9	NR (NR)	LT	35.2	10.5

a HA, hip arthroscopy; LT, long-term; MT, mid-term; NR, not reported. OA, osteoarthritis; ST, short term; THA, total hip arthroscopy.

### Conversion to THA/Hip Resurfacing

#### Meta-analysis

**Figure 4.**
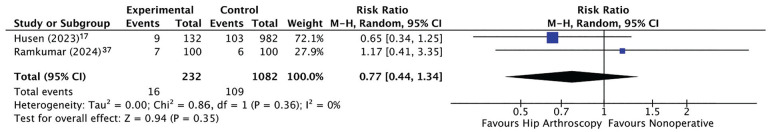
A forest plot demonstrating the risk ratio for conversion to THA/hip resurfacing with HA versus nonoperative management. M-H, Mantel-Haenszel; HA, hip arthroscopy; RR, risk ratio; THA, total hip arthroscopy.

Two LT follow-up comparative studies compared conversion to THA/hip resurfacing.^17,37^ One study^37^ included patients who underwent hip resurfacing along with those who underwent THA for their calculations. There was a reduced risk of conversion to THA/hip resurfacing with HA; however, this was not statistically significant (RR, 0.77 [95% CI, 0.44-1.34]; *P* = .35) (Figure 4). Therefore, patients with HA had 0.77 times the risk of conversion to THA/hip resurfacing compared with nonoperative treatment, or in other words, they had 23% less risk of progression of hip OA, but this was not statistically significant.

#### All Studies

There were 5 ST studies, with a range of conversion to THA from 0% to 13.2% (Table 4). One study^11^ found a statistically significant risk of conversion to THA for patients with baseline Tönnis grade 1 OA (6/18; 33%) compared with Tönnis grade 0 OA (1/35; 2.9%; *P* = .002). For MT studies, the rate of conversion was 2.4% to 29.7%. The study with the greatest conversion to THA was a comparative study that assessed patients who underwent HA for FAIS in patients with obesity and compared this with control patients without obesity.^35^ The mean BMI for these patients was 34.3 kg/m^2^, whereby the study with the next closest mean BMI of study patients was 25.6 kg/m^2^ (Table 1). If this obesity cohort was excluded, the range of conversion to THA at the MT follow-up would range from 0% to 7.5%. For LT operative follow-ups (4 studies), the conversion to THA ranged from 7% to 21.7%, and it ranged from 6% to 10.5% for nonoperative management. When solely assessing the 2 comparative studies assessing nonoperative management to operative management, the weighted rates of conversion to THA was 6.9% (SD, 0.1) in HA and 10.1% (SD, 1.4) in nonoperative patients.

**Table 4:** Reoperations^*a*^

			Conversion to THA/Hip Resurfacing					Revision Hip Arthroscopy				
Author (Year)		Hips, n	n	%	Mean Age, y	Mean Time to THA, mo	SD (Range)	n	%	Mean Age, y	Mean Time to Revision, mo	SD (Range)
*Short-term*												
Domb et al^7^ (2013)		22	0	0				2	9.1		17.5	NR (8-27)
Gicquel et al^11^ (2014)		53	7	13.2		28						
Hartigan et al^15^ (2016)		16	1	6.3		6						
Hufeland et al^16^ (2016)		44	5	11.4		28	7.1 (12-56)					
Mardones et al^25^ (2016)		18						1	5.6		NR	NR
Palmer et al^33^ (2012)		201	13	7.5		17.7	NR (2-46)					
Perets et al^34^ (2017)		41						3	7.3		25.1	NR
*Mid-term*												
Haefeli et al^13^ (2017)		52	2	3.8		96.0	NR (84-108)	9	17.0		NR	NR
Kierkegaard et al^18^ (2022)		60	6	10	46			12	20.0		NR	NR
Lee et al^22^ (2019)		41	1	2.4		86		5	12.2		26.6	NR (15-49)
Perets et al^35^ (2018)^3^												
	HA, Obesity	74	22	29.7		30.6	21.9 (3-73)	6	8.1		19.3	14.9 (7-48)
	HA, control	74	11	14.9		35.7	19.2 (4-60)	8	10.8		27.8	18.8 (4-56)
*Long-term*												
Domb et al^3^ (2023)												
	HA, repair	46	5	10.9		93.8	28.2 (NR)	5	10.9		33.4	32.6 (NR)
	HA, debridement	46	10	21.7		52.8	38.2 (NR)	3	6.5		43.6	64.1 (NR)
Husen et al^17^ (2023)^1^												
	HA	132	9	6.8								
	Nonoperative	982	103	10.5								
Más Martínez et al^26^ (2024)^2^		71	13	18.3	50	63		4	5.6	42.2	39	
Ramkumar et al^37^ (2024)												
	HA	100	7	7	40							
	Nonoperative	100	6	6	40							

a HA, hip arthroscopy; NR, not reported; THA, total hip arthroplasty.

### Risk Factors for Conversion to THA/Hip Resurfacing

For operative management, 2 studies^16,18^ found age to be a statistically significant risk factor for conversion to THA. Three studies^11,16,17^ found higher baseline OA grades to be a statistically significant risk factor for conversion to THA. One study^35^ found obesity to be a risk factor for conversion to THA. For nonoperative management, 1 study^17^ found baseline OA, male sex, and cam morphology to be statistically significant risk factors for conversion to THA. There were no clear trends with pre- or postoperative alpha angles and conversion to THA/hip resurfacing.

### Revision Arthroscopy

The revision arthroscopy rates were 5.6% to 9.1% in ST studies, 8.1% to 20.0% in MT studies, and 5.6% to 10.9% in LT studies (Table 4). Indications for revision HA included persistent/recurrent pain, capsulolabral adhesions, suture pullout, insufficient femoral osteoplasty, and heterotopic ossification.^7,22,26^ No study reported revision arthroscopy specifically for OA progression. One study^13^ found that a preoperative lateral center edge angle of >33°, a Tönnis angle of <3°, and a pre- or postoperative pistol grip deformity were associated with revision arthroscopy.

### Labral Repair versus Labral Debridement

Three studies^3,7,18^ reported OA progression in cohorts where ≥95% of patients underwent labral repair, with 1 study^3^ directly comparing all patients who underwent labral repair with all patients undergoing debridement. No additional studies had cohorts of ≥95% of patients who underwent labral debridement. For patients undergoing labral repair, rates of OA progression were 8.7% to 23%. Of note, the study conducted by Kierkegaard et al,^18^ with the highest progression of OA, had 2 patients (5%) with Tönnis grade 2 OA. With this study removed, the rate of OA progression was 8.7% to 13.6%. For labral debridement, OA progression was reported as 4.3%. However, the rate of conversion to THA ranged from 0% to 10.9% for labral repair and was reported as 21.7% for labral debridement. In the comparative study,^3^ there were no statistically significant differences in survivorship between groups, although patients with labral debridement underwent conversion to THA at significantly earlier time points (*P* = .048).

### Patient-Reported Outcome Measures

All commonly reported PROMs can be found in Supplemental Content 5. Nine studies^††^ reported pre- and postoperative mHHS scores, with a mean postoperative score ranging from 78.5 to 95.8. Six studies^7,15,22,26,39^ reported pre- and postoperative HOS acts of daily living scores, and 4 studies^7,15,22,26^ found statistically significant improvements. Seven studies^7,15,22,26,34,35,39^ reported baseline and follow-up HOS sport-specific subscale scores, and 5 studies^7,15,22,26,34^ found statistically significant improvements. Non-Arthritic Hip Scores were reported in 5 studies^7,15,33-35^; 4 studies^7,15,33,34^ reported statistically significant improvements. VAS-Pain scores were reported in 8 studies^7,15,22,25,33-35,39^, with mean follow-up scores ranging from 1.7 to 2.9, and 5 studies^7,15,22,33,34^ reported statistically significant improvements. Five studies^7,11,22,34,35^ reported patient satisfaction that ranged from 7.6 to 8.4 out of 10 for patients who had HA. No PROMs were reported for nonoperative patients.

## Discussion

The primary findings of this review are as follows: (1) Radiographic hip OA progression appears to increase over time after HA, and (2) there is evidence, although limited, of reduced radiographic OA progression (any increase in grading) for patients who undergo HA for FAIS at LT follow-ups (>10 years). Although there were only 2 comparative studies^17,37^ that compared operative and nonoperative management, there was a 32% (*P* = .002) less risk of radiographic OA progression (any increase in grading) with HA compared with nonoperative management. However, there were significant differences in sample sizes, with a combined 232 hips undergoing HA compared to 1082 nonoperative hips, which may introduce additional bias. Additionally, there was a nonsignificant (RR, 0.77; *P* = .35) decreased risk of conversion to THA/hip resurfacing with operative management of FAIS. After HA, risk factors for conversion to THA included increased age, baseline OA, and obesity. With nonoperative management, baseline OA, male sex, and cam morphology were risk factors for conversion to THA. There was also an increasing trend of progression of hip OA with longer follow-up durations.

HA may reduce the risk of progression of hip OA by addressing the premature contact of the femoral head/neck with the acetabulum as seen with patients with cam morphology. Husen et al^17^ found that for nonoperative patients with a cam morphology, they had a hazard ratio of 3.5 (*P* < .01) for conversion to THA compared with those without. Cam morphology can be treated with femoral osteochondroplasty, and the reshaping of the femur may limit the mechanical wear caused by the premature impact; however, there is limited research demonstrating that cam resection in itself reduces the risk of hip OA progression. The present study did find a trend of worsening radiographic OA progression with larger preoperative/baseline alpha angles, although this was not as clear when assessing postoperative alpha angles (Table 3). Furthermore, these findings are significantly limited by confounding follow-up durations. Therefore, larger baseline cam morphology may cause more significant preexisting damage to the acetabulum cartilage and have a greater impact on the overall progression of radiographic OA. This is in keeping with the pattern of chondrolabral junction (CLJ) wear associated with cam morphology FAIS, whereas patients suffering from pincer morphologies who have more intrasubstance labral tearing do not have such reported correlations, at least with respect to conversion to THA.^1,17^

For patients with cartilage defects, options for addressing lesions include debridement, microfracture, autologous chondrocyte implantation (ACI), matrix-induced ACI (MACI), osteochondral autologous transplantation (OATS), and osteochondral allograft transfer (OCA).^45^ Six included studies^3,18,25,33-35^ documented the use of microfracture in some proportion of patients. Domb et al^6^ found that even for patients with full-thickness chondral defects that required microfracture, their ST follow-up PROMs were not significantly different from those who did not have defects requiring microfracture. As such, microfracture may be an effective, at least in the short-term, method for addressing full-thickness chondral defects.^4,6^ In addition, Gicquel et al^11^ reported no differences in the progression of OA with patients who had cartilage procedures (microfracture). No included studies reported specifically on the use or impact of ACI, MACI, OATS, or OCA on the progression of OA.

The ability to address the labrum is another possibility that may impact radiographic hip OA progression. Dean et al^2^ found that patients who underwent HA with a more advanced breakdown of the CLJ/cartilage had a significant association with conversion to THA. In addition, when considering labral repair versus debridement, Domb et al^3^ found that patients undergoing HA with labral debridement had significantly earlier conversions to THA when compared with repair. Labral reconstruction is another option for patients with irreparable labral tears that can restore the suction seal of the hip and improve patient outcomes.^40^ The present review excluded patients who underwent labral reconstruction to reduce the heterogeneity of included studies. For patients with labral tears secondary to FAIS, a previous study demonstrated that the risk of conversion to THA was similar for labral repair and labral reconstruction, which may indicate at least similar impacts between the 2 on the progression of hip OA.^40^ Furthermore, although not examined in the present study, patients with hip dysplasia are particularly predisposed to labral injuries secondary to acetabular undercoverage, which is an additional risk factor for OA development.^27^

The present study did not find a statistically significant reduction in conversion to THA/hip resurfacing after HA when compared with nonoperative management. Hip resurfacing was reported in 2 studies^3,37^ combined with conversion to THA rates, as it is a similar option for younger and more active patients.^30^ Therefore, it may be a combination of factors that lead to reduced progression of hip OA but not to reduced rates of THA. This may be due to risk factors for conversion to THA that are not modifiable with or without HA, as well as the possibility of patients undergoing hip arthroplasty for other reasons than worsened radiographic OA (ie, worsened symptoms without radiographic progression, inflammatory causes, capsular or labral insufficiency, etc). In particular, patients with baseline OA have greater cartilage wear and smaller joint spaces, which is in itself an indication for a THA, and therefore, understandably have higher proportions of conversions to THA than those with preserved joint spaces.^5^ One included study^11^ did find that patients with a preoperative Tönnis grade of 1 compared with a Tönnis grade of 0 was a statistically significant predictor of OA progression (*P* = .046) and conversion to THA (*P* = .002). Two studies^16,17^ also found higher baseline OA grades to be a statistically significant risk factor for conversion to THA. Husen et al^17^ found that patients with no radiographic signs of OA had better survival rates than those with Tönnis grades ≥1 (*P* < .01). Hufeland et al^16^ also found a significantly higher mean KL grade for those who converted to THA, 2.6 compared with 2 (*P* < .01), respectively.

Advanced age and obesity are 2 other risk factors that are not addressed during HA. Two studies^16,18^ found increasing age to be a statistically significant risk factor for conversion to THA. Hufeland et al^16^ found that the mean age for patients converting to THA was 49.8 years, compared with a mean age of 33 years for those who did not. Similarly, Kierkegaard et al^18^ found a mean age of 46 years for those undergoing THA compared with 36 years in those who did not. When assessing obesity, Perets et al^35^ found that conversion to THA rates were higher in the group with obesity (27.9%) compared with the control group (14.9%; *P* = .04). Therefore, although HA may reduce the risk of radiographic OA progression, it may not result in decreased conversion to THA because of unmodified risk factors.

When assessing the reported progression of hip OA, the included studies were stratified based on follow-up periods, as OA worsens over time.^8^ Unsurprisingly, there was an overall increase in the trend of progression of hip OA with increasing the follow-up period (Figure 3). However, there were some outliers to this overall trend. The study with the greatest reported progression of OA with HA was conducted by Gicquel et al,^11^ with a rate of 37.1% with ST follow-up. This study had a few significant limitations that may account for the high progression of the OA rate. (1) The study included patients who had surgery from March 2008 to March 2009, whose surgeries may not have been done with the most modern HA methods. (2) Of the initial hips enrolled, only 35 of 53 hips were assessed for the progression of OA (34% lost to follow-up). (3) Of the 13 patients who had progression of OA, 7 were included in this group because of conversion to THA and not because of specifically radiographic progression, which is confounding as there are multiple other indications for THA.^11^ One study by Kierkegaard et al^18^ had a particularly high rate of OA progression for MT follow-up (23%). This higher rate of progression was potentially caused by a few factors. They had a high loss to radiographic follow-up (23%), and they did not have Tönnis grades for all baseline patients (39/60). These methodological challenges and baseline population differences may have accounted for the significantly higher rate of OA progression, but again, the overall trend was for worsening OA progression with time.

### Limitations

There are some limitations in our review. Not all studies reported individual patient changes in Tönnis grading and instead reported the baseline and follow-up cohort grades, which may underestimate the number of patients who had radiographic progression of OA. In addition, some papers excluded patients who underwent THA from their follow-up Tönnis grades. However, for a more accurate appreciation of OA progression, these patients were included in our analysis as having Tönnis grade 3 OA. By choosing to grade all patients who underwent THA as having grade 3, this may be an overestimation of the degree of OA progression.^26^ Tönnis grading also has limitations with inter-rater reliability, leading to additional possibilities for error when reporting grades.^36,41^ Furthermore, only 2 studies^17,37^ compared the progression of hip OA between operative and nonoperative management, with 1 study including vastly more hips in the nonoperative group (982 compared with 100), thereby weighting the results vastly toward this study.^17^ The vast majority (12 studies^3,7,11,13,15,16,18,22,25,26,34,39^) had <100 patients, and 7 studies^7,13,16,22,25,34,39^) had ≤50 patients; however, there was no clear association between smaller sample sizes and risk of bias. Lastly, most included studies were retrospective studies and case series, which could introduce significant bias into patient selection and outcomes.

## Conclusion

Our systematic review demonstrated that studies of patients undergoing HA for FAIS appear to have increasing radiographic progression of hip OA over time. Although significantly limited by only 2 retrospective cohort studies, subgroup analysis comparing operative versus nonoperative management demonstrated a 32% reduction in the radiographic progression of OA (any increase in grading) at LT follow-up. However, there were no significant differences in the risk of THA/hip resurfacing. Future long-term, high-level controlled studies are needed to help further understand this important clinical question.

## Supplemental Material

sj-docx-1-ojs-10.1177_23259671251326116 – Supplemental material for The Impact of Hip Arthroscopy on the Progression of Hip Osteoarthritis in Patients With Femoroacetabular Impingement Syndrome: A Systematic Review and Meta-analysis Supplemental material, sj-docx-1-ojs-10.1177_23259671251326116 for The Impact of Hip Arthroscopy on the Progression of Hip Osteoarthritis in Patients With Femoroacetabular Impingement Syndrome: A Systematic Review and Meta-analysis by Darius L. Lameire, Ananya Pathak, Shu Yang Hu, Yue Ting Kero Yuen, Daniel B. Whelan, Tim Dwyer, Tyler M. Hauer and Jaskarndip Chahal in The Orthopaedic Journal of Sports Medicine

## Footnotes

# References 3, 7, 11, 13, 15-18, 22, 25, 26, 33-35, 37, 39.

** References 7, 13, 15, 16, 18, 22, 25, 26, 33, 34.
