# Supplementary material for: The Impact of Hip Arthroscopy on the Progression of Hip Osteoarthritis in Patients With Femoroacetabular Impingement Syndrome: A Systematic Review and Meta-analysis
Source: Orthop J Sports Med. 2025 Apr 2;13(4):23259671251326116. doi: 10.1177/23259671251326116 (PMC11967232; doi:10.1177/23259671251326116)
Supplement: sj-docx-1-ojs-10.1177_23259671251326116 – Supplemental material for The Impact of Hip Arthroscopy on the Progression of Hip Osteoarthritis in Patients With Femoroacetabular Impingement Syndrome: A Systematic Review and Meta-analysis [file sj-docx-1-ojs-10.1177_23259671251326116.docx]

Supplemental Content 1: Search Strategy

| Medline | Embase | ClinicalTrials.gov |
| --- | --- | --- |
| **1**  exp Hip Joint/ or exp Osteoarthritis, Hip/ or exp Hip/ or hip*.mp. (487684)  **2**  exp Femoracetabular Impingement/ or femoroacetabular impingement.mp. or femo*.mp. (209641)  **3**  exp Femur/ or femur.mp. (103561)  **4**  1 or 2 or 3 (683192)  **5**  exp Arthroscopy/ or arthroscop*.mp. (46543)  **6**  osteochondro*.mp. (13937)  **7**  osteochondroplasty.mp. (258)  **8**  labr*.mp. (14792)  **9**  exp Arthritis/ or arthritis.mp. or osteoarthritis/ (370760)  **10**  tonnis.mp. (952)  **11**  kellgren.mp. (3864)  **12**  exp Osteoarthritis, Hip/ or lawrence.mp. (16485)  **13**  5 or 6 or 7 or 8 (71199)  **14**  9 or 10 or 11 or 12 (375402)  **15**  4 and 13 and 14 (1776) | **1**  exp Hip Joint/ or exp Osteoarthritis, Hip/ or exp Hip/ or hip*.mp. (751308)  **2**  exp Femoracetabular Impingement/ or femoroacetabular impingement.mp. or femo*.mp. (308107)  **3**  exp Femur/ or femur.mp. (193271)  **4**  1 or 2 or 3 (1049894)  **5**  exp Arthroscopy/ or arthroscop*.mp. (63478)  **6**  osteochondro*.mp. (12914)  **7**  osteochondroplasty.mp. (350)  **8**  labr*.mp. (18404)  **9**  exp Arthritis/ or arthritis.mp. or osteoarthritis/ (675480)  **10**  tonnis.mp. (1231)  **11**  kellgren.mp. (7523)  **12**  exp Osteoarthritis, Hip/ or lawrence.mp. (28472)  **13**  5 or 6 or 7 or 8 (89564)  **14**  9 or 10 or 11 or 12 (682629)  **15**  4 and 13 and 14 (4048) | **1**  (hip or femur or femo* or FAI) and (scope or arthroscopy or osteochondroplasty or labral) and (arthritis or osteoarthritis or tonnis or arthropathy) (359) |

Supplemental Content 2: Study Characteristics

| **Author (Year)** | **Title** | **Journal** | **Published Year** | **Recruitment Period** | **Country** | **Follow Up Period** | **Study Design** | **LOE** | **Group 1** | **Group 2** | **MINORS** |
| --- | --- | --- | --- | --- | --- | --- | --- | --- | --- | --- | --- |
| Domb (2013) | Arthroscopic capsular plication and labral preservation in borderline hip dysplasia: two-year clinical outcomes of a surgical approach to a challenging problem | AJSM | 2013 | Apr 2008 - Nov 2010 | USA | Short Term | Case Series | IV | Hip arthroscopy |  | 13 |
| Domb (2023) | Ten-Year Survivorship, Outcomes, and Sports Participation in Athletes After Primary Hip Arthroscopy for Femoroacetabular Impingement Syndrome | AJSM | 2023 | Feb 2008 - Dec 2010 | USA | Long Term | Retrospective Cohort | III | Hip arthroscopy - labral repair | Hip arthroscopy - labral debridement | 22 |
| Gicquel (2014) | Function and osteoarthritis progression after arthroscopic treatment of femoro-acetabular impingement: A prospective study after a mean follow-up of 4.6 (4.2–5.5) years | Orthop Traumatol Surg Res | 2014 | Mar 2008 - Mar 2009 | France | Short Term | Prospective Cohort | IV | Hip arthroscopy - Tӧnnis Grade 0 | Hip arthroscopy - Tӧnnis Grade 1 | 20 |
| Haefeli (2017) | What Are the Risk Factors for Revision Surgery After Hip Arthroscopy for Femoroacetabular Impingement at 7-year Followup? | Clin Orthop & Rel Res | 2017 | 2003 - 2008 | Switzerland | Mid Term | Case Series | IV | Hip arthroscopy |  | 13 |
| Hartigan (2016) | Clinical Outcomes of Hip Arthroscopy in Radiographically Diagnosed Retroverted Acetabula | AJSM | 2016 | Jun 2008 - Feb 2012 | USA | Short Term | Case Series | IV | Hip arthroscopy |  | 13 |
| Hufeland (2016) | Arthroscopic treatment of femoroacetabular impingement shows persistent clinical improvement in the mid-term | Arch Orthop Trauma Surg | 2016 | Mar 2004 - Dec 2007 | Germany | Short Term | Case Series | IV | Hip arthroscopy |  | 12 |
| Husen (2023) | Progression of Osteoarthritis at Long-term Follow-up in Patients Treated for Symptomatic Femoroacetabular Impingement With Hip Arthroscopy Compared With Nonsurgically Treated Patients | AJSM | 2023 | 1998 - 2015 | USA | Long Term | Retrospective Cohort | III | Hip arthroscopy | Nonoperative treatment | 21 |
| Kierkegaard (2022) | Five-Year Follow-up After Hip Arthroscopic Surgery in the Horsens-Aarhus Femoroacetabular Impingement (HAFAI) Cohort | OJSM | 2022 | 2015 - 2016 | Denmark | Mid Term | Case Series | IV | Hip arthroscopy |  | 13 |
| Lee (2019) | Arthroscopic repair of acetabular labral tears associated with femoroacetabular impingement: 7-10 years of long-term follow-up results | CiOS | 2019 | Jan 2008 - Dec 2010 | Korea | Mid Term | Case Series | IV | Hip arthroscopy |  | 12 |
| Mardones (2016) | Arthroscopic release of iliopsoas tendon in patients with femoro-acetabular impingement: clinical results at mid-term follow-up | Musc, Lig & Tendons | 2016 | May 2011- Dec 2012 | Chile | Short Term | Case Series | IV | Hip arthroscopy |  | 12 |
| Más Martinez (2024) | Hip arthroscopy for femoroacetabular impingement with 10-year minimum follow-up | Rev Esp Cir Ortop Traumatol | 2024 | Jan 2010 - Dec 2011 | Spain | Long Term | Case Series | IV | Hip arthroscopy |  | 14 |
| Palmer (2012) | Midterm outcomes in patients with cam femoroacetabular impingement treated arthroscopically | Arthroscopy | 2012 | May 2005 - May 2008 | USA | Short Term | Case Series | IV | Hip arthroscopy |  | 13 |
| Perets (2017) | Outcomes of Hip Arthroscopy in Competitive Athletes | Arthroscopy | 2017 | Feb 2008 - Nov 2013 | USA | Short Term | Case Series | IV | Hip arthroscopy |  | 14 |
| Perets (2018) | Minimum five-year outcomes of hip arthroscopy for the treatment of femoroacetabular impingement and labral tears in patients with obesity: A match-controlled study | JBJS | 2018 | Feb 2008 - Dec 2010 | USA | Mid Term | Retrospective Cohort | III | Hip arthroscopy - patients with obesity | Hip arthroscopy - control patients | 20 |
| Ramkumar (2024) | Modern Hip Arthroscopy for FAIS May Delay the Natural History of Osteoarthritis in 25% of Patients: A 12-Year Follow-up Analysis | AJSM | 2024 | 2010 - 2012 | USA | Long Term | Retrospective Cohort | III | Hip arthroscopy | Nonoperative treatment | 20 |
| Rhee (2016) | Clinical outcomes after arthroscopic acetabular labral repair using knot-tying or knotless suture technique | Arch Orthop Trauma Surg | 2016 | Sep 2012 - May 2013 | South Korea | Short Term | RCT | II | Hip arthroscopy - knot tying | Hip arthroscopy - knotless | NA |

LOE: Level of evidence; MINORS: methodological index for non-randomized studies; RCT: randomized controlled trial

Supplemental Content 3: Risk of Bias Table


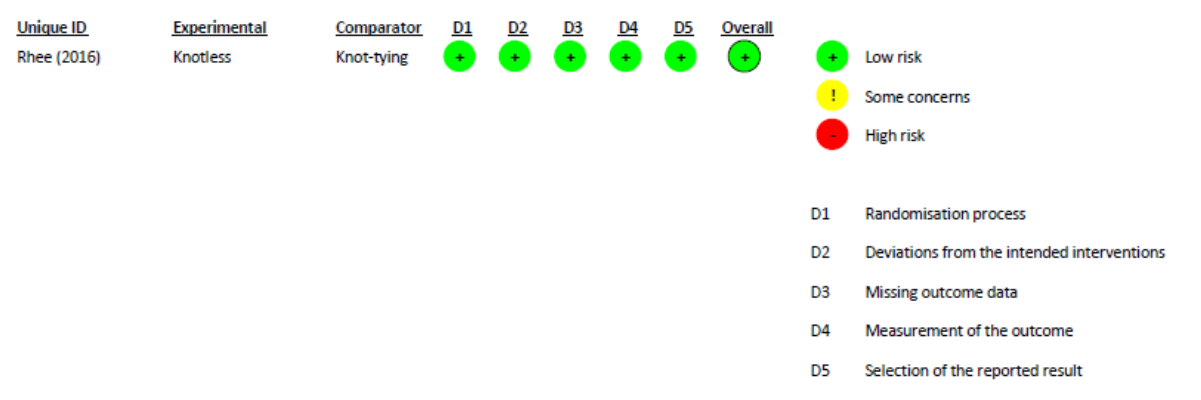


Supplemental Content 4: Proportion of Radiographic OA Worsening

|  |  | **Baseline Tӧnnis Grade, %** | | | | **Follow Up Tӧnnis Grade, %** | | | | **Progression** |
| --- | --- | --- | --- | --- | --- | --- | --- | --- | --- | --- |
| **Author (Year)** |  | 0 | 1 | 2 | 3 | 0 | 1 | 2 | 3 | Proportion, % |
| ***Short-Term*** |  |  |  |  |  |  |  |  |  |  |
| Hartigan (2016) |  | 86.7 | 13.3 |  |  | 81.3 | 12.5 |  | 6.3 | 6.3 |
| Mardones (2016) |  | 58.8 | 41.2 |  |  | 58.8 | 41.2 |  |  | 0.0 |
| Perets (2017) |  | 100.0 |  |  |  | 97.6 | 2.4 | 0.0 |  | 2.4 |
| ***Mid-Term*** |  |  |  |  |  |  |  |  |  |  |
| Haefeli (2017) |  | 82.7 | 17.3 |  |  | 54.8 | 41.9 | 3.2 |  | 27.9 |
| Kierkegaard (2022) |  | 74.4 | 20.5 | 5.1 |  | 46.5 | 37.2 | 7.0 | 9.3 | 27.8 |
| Perets (2018) |  |  |  |  |  |  |  |  |  |  |
|  | HA - Obesity | 66.2 | 33.8 |  |  | 63.5 | 33.8 | 1.4 | 1.4 | 2.7 |
|  | HA - Control | 70.3 | 29.7 |  |  | 70.3 | 29.7 |  |  | 0.0 |
| ***Long-Term*** |  |  |  |  |  |  |  |  |  |  |
| Domb (2023) |  |  |  |  |  |  |  |  |  |  |
|  | HA - Repair | 80.4 | 19.6 |  |  | 71.7 | 28.3 |  |  | 8.7 |
|  | HA - Debridement | 67.4 | 32.6 |  |  | 63.0 | 37.0 |  |  | 4.3 |
| Husen (2023) |  |  |  |  |  |  |  |  |  |  |
|  | HA | 32.6 | 65.2 | 2.3 |  | 20.5 | 61.4 | 12.1 | 6.1 | 15.9 |
|  | Nonoperative | 35.6 | 58.0 | 5.7 | 0.6 | 21.3 | 51.6 | 21.9 | 5.2 | 20.8 |
| Más Martinez (2024) |  | 32.4 | 32.4 | 22.5 | 12.7 | 32.4 | 31.0 | 11.3 | 25.4 | 12.7 |
| Ramkumar (2024) |  |  |  |  |  |  |  |  |  |  |
|  | HA | 78.0 | 20.0 | 2.0 |  | 57.0 | 34.0 | 2.0 | 7.0 | 21.0 |
|  | Nonoperative | 73.0 | 26.0 | 1.0 |  | 38.0 | 45.0 | 9.0 | 8.0 | 35.0 |

HA: hip arthroscopy

Supplemental Content 5: Patient Reported Outcome Measures

|  |  | **mHHS** | | | | | **HOS-ADL** | | | | | **HOS-SSS** | | | | | **NAHS** | | | | | **VAS** | | | | | **Patient Satisfaction** | |
| --- | --- | --- | --- | --- | --- | --- | --- | --- | --- | --- | --- | --- | --- | --- | --- | --- | --- | --- | --- | --- | --- | --- | --- | --- | --- | --- | --- | --- |
|  |  | **Preoperative** | | **Postoperative** | |  | **Preoperative** | | **Postoperative** | |  | **Preoperative** | | **Postoperative** | |  | **Preoperative** | | **Postoperative** | |  | **Preoperative** | | **Postoperative** | |  |  |  |
| **Author (Year)** |  | **Mean** | **SD (Range)** | **Mean** | **SD (Range)** | **Pre/Post p-value** | **Mean** | **SD (Range)** | **Mean** | **SD (Range)** | **Pre/Post P-value** | **Mean** | **SD (Range)** | **Mean** | **SD (Range)** | **Pre/Post P-value** | **Mean** | **SD (Range)** | **Mean** | **SD (Range)** | **Pre/Post P-value** | **Mean** | **SD (Range)** | **Mean** | **SD (Range)** | **Pre/Post P-value** | **Mean** | **SD (Range)** |
| ***Short Term*** |  |  |  |  |  |  |  |  |  |  |  |  |  |  |  |  |  |  |  |  |  |  |  |  |  |  |  |  |
| Domb (2013) |  | 69.0 | 12.1 (NR) | 86.2 | 12.7 (NR) | <0.0001 | 72.9 | 12.3 (NR) | 89.6 | 12.3 (NR) | <0.0001 | 49.0 | 15.6 (NR) | 77.0 | 21.9 (NR) | <0.0001 | 68.6 | 11.7 (NR) | 85.9 | 14.2 (NR) | <0.0001 | 5.8 | 2.4 (NR) | 2.9 | 2.2 | <0.0001 | 8.4 | 1.4 (NR) |
| Hartigan (2016) |  | 65.4 |  | 81.1 |  | <0.001 | 68.8 |  | 88.4 |  | <0.001 | 47.3 |  | 76.4 |  | <0.001 | 64.7 |  | 85.7 |  | <0.001 | 5.9 |  | 2.5 |  | <0.001 |  |  |
| Hufeland (2016) |  | 67.2 | 6.4 (NR) | 86.4 | 13.5 (NR) | <0.001 |  |  |  |  |  |  |  |  |  |  |  |  |  |  |  |  |  |  |  |  |  |  |
| Mardones (2016) |  | 74.7 | 17 (40-94) | 95.8 | 8.4 (69-100) | <0.001 |  |  |  |  |  |  |  |  |  |  |  |  |  |  |  | 5.5* | NR (3-7) | 0* | (0-5) |  |  |  |
| Palmer (2012) |  |  |  |  |  |  |  |  |  |  |  |  |  |  |  |  | 56.1 | 15.9 (NR) | 78.2 | 15.8 (NR) | <0.001 | 6.8 | 1.9 (NR) | 2.7 | 2.2 | <0.001 |  |  |
| Perets (2017) |  | 67.1 | 13.3 (NR) | 83.5 | 11.3 (NR) | <0.0001 |  |  |  |  |  | 46.9 | 23.4 (NR) | 80.1 | 22.9 (NR) | <0.0001 | 66.8 | 18.7 (NR) | 88.8 | 18.5 (NR) | <0.0001 | 5.1 | 2.4 (NR) | 1.7 | 2.1 | <0.0001 | 7.5 | 2.7 (NR) |
| Rhee (2016) |  |  |  |  |  |  |  |  |  |  |  |  |  |  |  |  |  |  |  |  |  |  |  |  |  |  |  |  |
|  | HA - Knot-tying | 66.6 | 13.4 (NR) | 78.5 | 13.5 (NR) |  | 50.9 | 13.7 (NR) | 63.1 | 7.8 (NR) |  | 66.9 | 17.1 (NR) | 75.2 | 21.3 (NR) |  |  |  |  |  |  | 5.9 | 2.5 (NR) | 2.3 | 1.8 |  |  |  |
|  | HA - Knotless | 66.0 | 19.7 (NR) | 84.8 | 9.2 (NR) |  | 51.3 | 17.7 (NR) | 59.9 | 12.4 (NR) |  | 58.0 | 19.9 (NR) | 70.0 | 27.0 (NR) |  |  |  |  |  |  | 6.4 | 1.5 (NR) | 2.9 | 2.2 |  |  |  |
|  |  |  |  |  |  |  |  |  |  |  |  |  |  |  |  |  |  |  |  |  |  |  |  |  |  |  |  |  |
| ***Mid Term*** |  |  |  |  |  |  |  |  |  |  |  |  |  |  |  |  |  |  |  |  |  |  |  |  |  |  |  |  |
| Lee (2019) |  | 59.5 | NR (38-82) | 86.8 | NR (61-95.7) | <0.01 | 58.3 | NR (20-80) | 85.2 | NR (45-95) | <0.01 | 51.2 | NR (10-80) | 82.4 | NR (50-95) | <0.01 |  |  |  |  |  | 6.4 |  | 1.8 |  | <0.001 | 7.6 | 2.2 (NR) |
| Perets (2018) |  |  |  |  |  |  |  |  |  |  |  |  |  |  |  |  |  |  |  |  |  |  |  |  |  |  |  |  |
|  | HA - Obesity | 51.8 | 13.1 (NR) | 78.6 | 20.7 (NR) |  |  |  |  |  |  | 25.2 | 21.3 (NR) | 62.9 | 30.8 (NR) |  | 45.1 | 17.6 (NR) | 76.9 | 21.7 (NR) |  | 6.9 | 1.8 (NR) | 2.9 | 2.9 |  | 8.3 | 2.0 (NR) |
|  | HA - Control | 60.9 | 15.2 (NR) | 82.7 | 16.0 (NR) |  |  |  |  |  |  | 37.7 | 26.5 (NR) | 70.0 | 24.7 (NR) |  | 58.4 | 17.2 (NR) | 83.3 | 13.6 (NR) |  | 5.9 | 1.9 (NR) | 2.4 | 2.3 |  | 7.9 | 2.4 (NR) |
|  |  |  |  |  |  |  |  |  |  |  |  |  |  |  |  |  |  |  |  |  |  |  |  |  |  |  |  |  |
| ***Long Term*** |  |  |  |  |  |  |  |  |  |  |  |  |  |  |  |  |  |  |  |  |  |  |  |  |  |  |  |  |
| Más Martinez (2024) |  | 70.4 | 14.2 (NR) | 93.0 | 9.8 (NR) | <0.001 | 62.0 | 16.6 (NR) | 88.6 | 14.3 (NR) | <0.001 | 35.5 | 24.8 (NR) | 74.1 | 28.8 (NR) | <0.001 |  |  |  |  |  |  |  |  |  |  |  |  |

*denotes median; mHHS: modified Harris Hip Score; HOS-ADL: Hip Outcome Score – Acts of Daily Living; HOS-SSS: Hip Outcome Score – Sport-Specific Subscale; NAHS: Non Arthritic Hip Score; NR: Not reported; VAS – Visual Analog Scale.
